# The Siblings with Blistered Legs: Marking Nut Maladies in India

**DOI:** 10.4269/ajtmh.21-0929

**Published:** 2021-11-15

**Authors:** Jami Rupa Ramani, Hima Gopinath, Prabhakaran Nagendran

**Affiliations:** Department of Dermatology, All India Institute of Medical Sciences, Mangalagiri, Andhra Pradesh, India

**Figure 1.**
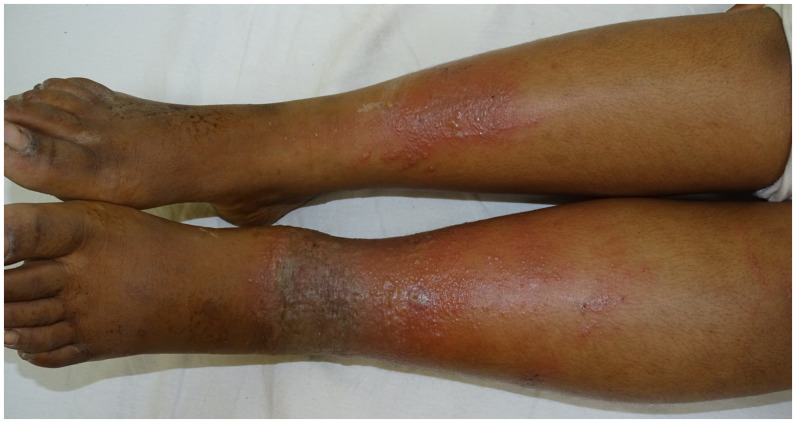
Erythema, edema, vesicles, and bullae over left ankle and lower leg with patchy dermatitis on right leg of girl. This figure appears in color at www.ajtmh.org.

**Figure 2.**
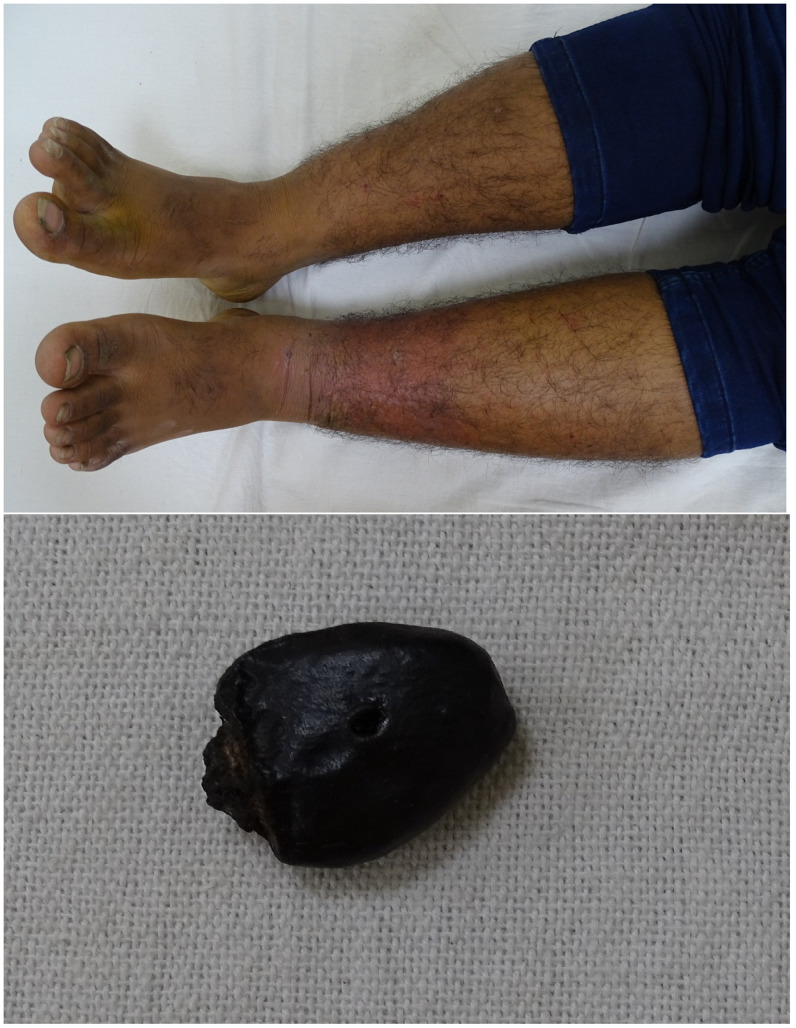
Circumferential erythema, edema, and vesicles over left ankle and lower leg of boy along with marking nut brought by the parents. This figure appears in color at www.ajtmh.org.

An 11-year-old girl and her 14-year-old brother presented with pain, itching, redness, and fluid-filled lesions on their legs for the past 3 days. On examination, both siblings had circumferential erythema, edema, vesicles, and bullae on the left ankle and lower leg (Figures 1 and 2). There was similar, but milder and patchy dermatitis on the right leg. The skin had a patchy yellow hue as a result of the application of turmeric on the day of the hospital visit. Five days ago, the parents punctured the marking nut and tied it with a thread around the left ankle of both siblings. They believed it would protect the siblings from the evil eye. The children were diagnosed to have marking nut dermatitis and were started on oral steroids and antihistamines. The symptoms had resolved at the 1-week follow-up visit.

The Indian marking nut (*Semecarpus anacardium*) has an important role in Indian traditional medicine and folklore. The brown-black juice of the nut has been used by dhobis (washermen) to leave an indelible mark on laundry. This had resulted in several cases of “dhobi-mark” or “marking-nut” dermatitis in British soldiers posted in India.^1^ This indigenous nut has been used in diverse situations including voodoo treatment, as an amulet, abortifacient, hair colorant, in the treatment of joint pains, alopecia areata, chronic skin conditions, and tattoo removal. It belongs to the genus *Toxicodendron* and the family Anacardiaceae. Several plants of this genus (such as poison ivy, poison sumac, poison oak, and the cashew nut tree) are infamous for contact dermatitis.^2^^,^^3^ The juice of the marking nut contains bhilawanol, a mixture of alkyl catechols, which is similar to other urushiols of the *Toxicodendron* genus. It can cause both irritant and allergic contact dermatitis. Ectopic contact dermatitis (transmitted through hands) in areas such as face and genitalia, airborne contact dermatitis, and spread through contaminated mail has also been reported. Clinical features can include pruritus, erythema, papules, urticaria, vesicles, bullae, periorbital edema, and erythema multiforme-like lesions. Nephrotoxicity may rarely occur because of systemic absorption.^1^^,^^4^ The history and awareness about the marking nut were crucial in diagnosing our patients. It is thus important to be aware of ethnobotany and cultural dermatoses in the tropics.
